# S-cone contribution to oscillatory potentials in patients with blue cone monochromacy

**DOI:** 10.1007/s10633-024-09981-y

**Published:** 2024-06-14

**Authors:** Giulia Righetti, Melanie Kempf, Susanne Kohl, Bernd Wissinger, Laura Kühlewein, Katarina Stingl, Krunoslav Stingl

**Affiliations:** 1 Center for Ophthalmology, University Eye Hospital, University of Tübingen, 72076 Tübingen, Germany; 2 Center for Rare Eye Diseases, University of Tübingen, 72076 Tübingen, Germany; 3 Molecular Genetics Laboratory, Center for Ophthalmology, Institute for Ophthalmic Research, University of Tübingen, 72076 Tübingen, Germany; 4 Institute for Ophthalmic Research, Center for Ophthalmology, University of Tübingen, Tübingen, Germany

**Keywords:** Blue cone monochromacy, Full-field ERG, Oscillatory potentials

## Abstract

**Purpose:**

The aim of this exploratory study is to investigate the role of S-cones in oscillatory potentials (OPs) generation by individuals with blue-cone monochromacy (BCM), retaining S-cones, and achromatopsia (ACHM), lacking cone functions.

**Methods:**

This retrospective study analyzed data from 39 ACHM patients, 20 BCM patients, and 26 controls. Central foveal thickness was obtained using spectral-domain optical coherence tomography, while amplitude and implicit time (IT) of a- and b-waves were extracted from the ISCEV Standard dark-adapted 3 cd.s.m^−2^ full-field ERG (ffERG). Time–frequency analysis of the same measurement enabled the extraction of OPs, providing insights into the dynamic characteristics of the recorded signal.

**Results:**

Both ACHM and BCM groups showed a significant reduction (*p* < .00001) of a- and b-wave amplitudes and ITs as well as the power of the OPs compared to the control groups. The comparison between ACHM and BCM didn’t show any statistically significant differences in the electrophysiological parameters. The analysis of covariance revealed significantly reduced central foveal thickness in the BCM group compared to ACHM and controls (*p* < .00001), and in ACHM compared to controls (*p* < .00001), after age correction and Tukey post-hoc analysis.

**Conclusions:**

S-cones do not significantly influence OPs, and the decline in OPs' power is not solely due to a reduced a-wave. This suggests a complex non-linear network influenced by photoreceptor inputs. Morphological changes don’t correlate directly with functional alterations, prompting further exploration of OPs’ function and physiological role.

## Introduction

Cones and rods are the two photoreceptor types contained in the human retina and both hyperpolarize in response to light. Rods are located mainly in the peripheral retina and mediate vision under dim light in scotopic conditions. Cones, in contrast, have their highest density mostly in the fovea and the macular region, support daylight or photopic vision, and require larger amounts of photons for excitation. In the human retina, there are three subtypes of cones each expressing a different photopigment enabling color vision. The S-, M- and L-cones express short (380–540 nm), medium (440–670 nm) and long (500–690 nm) wavelength-sensitive photopigments, respectively [1]. Compared with M- and L-cones, S-cones comprise only a small fraction (approximately 7%) of the total number of cones, reaching their maximum peak density at around 100–125 µm eccentricity from the fovea [2]. In terms of spatial distribution, S-cones are absent in the central fovea, creating an ‘S-free zone’ where visual function is mediated by L- and M-cones only [3,4]. Furthermore, it has been demonstrated that in electrophysiological recordings from S-cones, a- and b-waves are slower compared to L- and M-cones [5], although they are capable of responding to standard flicker stimuli [6] and contribute to the perception of motion [7].

Complete achromatopsia (ACHM, also known as rod monochromatism) and blue cone monochromacy (BCM) are two congenital cone dysfunction disorders affecting approximately one in 30.000 people and one in 100.000 people worldwide, respectively [8]. The underlying genetic causes of these diseases are well known. Specifically, for ACHM disease-causing variants in six different genes have been identified which account for more than 90% of clinically diagnosed cases. These genes comprise *CNGA3* [9] and *CNGB3* [10] (affecting together ~ 80% of cases [11,12]), *GNAT2* [13], *PDE6H* [14], *PDE6C* [15], and lastly *ATF6* [16]. BCM is an X-linked recessive trait and caused by mutations in the *OPN1LW* and *OPL1MW* genes on Xq28, which encode the apo-protein of the L- and M-cone photopigment, respectively [17,18]. Consequently, there is a functional loss of the L- and M-cones, while the functionality of the S-cones remains unaffected [19].

Clinically, ACHM and BCM share many features such as nystagmus, reduced visual acuity, color perception abnormalities and photophobia [20,21]. BCM individuals are often myopic and, due to the presence of intact S-cone function, they exhibit some residual color discrimination ability, although deterioration with increasing illumination has been reported [22]. Concurrently, ACHM individuals suffer from complete color blindness, lacking functional cones altogether. Vision in these individuals thereby relies solely on rod photoreceptors. In the rare incomplete subtype of ACHM, different extent of remaining cone function is observed. Albeit these cone function disorders are usually regarded as stationary diseases, previous studies applying imaging technologies indicate a progressive course especially in the structural integrity of cone outer segments [19,23,24].

The fundus usually appears normal in both diseases, although ACHM tends to exhibit fovea hypoplasia more frequently than in BCM individuals [25]. Alterations in foveal thickness were found with spectral-domain optical coherence tomography (SD-OCT) in both diseases. Previous studies on BCM reported a thinning of the central retina and specifically the outer layer of the photoreceptors, due to a shortening of outer and inner segments [26,27]. Thinning of the outer layer of photoreceptors has also been reported in ACHM in some studies [24,28,29], whereas Barthelmes and colleagues did not observe differences in comparison with normative population [26], which is usually the case in younger subjects. However, the relationship between age and cone loss is controversial [24,30]. With increasing age, the foveal degeneration progresses, leading to retinal thinning in later stages.

Full-field electroretinography (ffERG) under standard photopic conditions shows a marked reduction of cone activity or non-recordable responses in BCM but preservation of S-cone function if tested specifically [19,23]. In typical complete ACHM a complete absence of photoreceptor function is observed [29,31]. Under dark-adapted conditions, rod activity is usually normal or close to normal [29,31], although a decrease in scotopic responses has been reported in some patients [32]. To date, ffERG is a common, non-invasive tool widely used in visual electrophysiology for its ability to objectively measure activity originating from the retina [33]. By manipulating the type of light stimulus and its intensity, it is possible to measure different components of the retina, such as oscillatory potentials (OPs), in addition to the typical a- and b-waves that reflect the hyperpolarization of the photoreceptors [34], and the depolarization of the bipolar cells [35] and Müller cells [36], respectively. OPs are fast wavelets arising from the trough of the a-wave to peak of the b-wave [37,38]. Under scotopic conditions, they are elicited by the dark-adapted ERGs with a single flash at 3 or 10 cd.s.m^−2^ [39].

In clinic, alteration or suppression of OPs serve as markers of various retinal diseases, including diabetic retinopathy, high intra-ocular pressure, and central retinal vein occlusion [40,41]. The exact origin of OPs is under debate with different possible theories being proposed in the literature. The predominant hypothesis suggests that OPs result from interactions between multiple cells of the inner retinal layer, including amacrine, horizontal, and bipolar cells [42,43]. Specifically, some researchers highlight the significance of synaptic connections between the rod bipolar axon terminals and amacrine cells [44], while others propose involvement of ganglion cells [43,45]. In a previous study, we compared OPs between individuals with ACHM and controls, showing that OPs are significantly reduced in the first, but nevertheless present [46]. These findings emphasize the importance of a functioning cone pathway to the formation of the dark-adapted OPs.

Given the basic functional difference between ACHM and BCM, the purpose of this exploratory study was to investigate how the presence of S-cones contributes to the integrity of the cone pathway. Therefore, we examined the presence of possible electrophysiological markers using ffERG, by looking at relationships between a- and b-wave components and OPs, with the aim of estimating a possible contribution of S-cones on the latter. Moreover, we investigated how S-cones in patients with BCM impact the structure of the retina in relationship with age and OPs.

## Methods

### Participants and setup

This study retrospectively analyzed data from full-field electroretinography (ffERG, ColorDome stimulator and the Espion E2 software, Diagnosys Ltd., Cambridge, UK) according to the ISCEV standards [39] and spectral-domain optical coherence tomography (SD-OCT, Heidelberg Engineering GmbH, Heidelberg, Germany) from 39 genetically confirmed ACHM patients (comprising of 20 individuals with bi-allelic variants in *CNGA3*-ACHM, 17 in *CNGB3*-ACHM and 1 in *GNAT2*-ACHM; mean age 35.6, ± 11), 20 genetically confirmed BCM patients (mean age 36.5, ± 18) and 26 controls (mean age 40.1, ± 11) volunteers.

The standard operating procedure in our diagnostic set-up uses silver corneal fiber electrodes (DTL-electrode, Diagnosys Ltd., Dublin, Ireland) that are used as positive electrodes and gold cup as a reference. Ground electrodes are placed on the forehead, respectively. The analyses were only performed on the dark-adapted 3 cd.s.m^−2^ flash response data (DA 3, as per ISCEV [39]). The choice of this dark-adapted measurement is based on the fact that the presented stimulus elicits responses from both cones and rods. This protocol also enables a detailed observation of the generation of the oscillatory potentials (OPs) [39]. A single trace was extracted for each participant from the average of five results, to which a bandpass filter was applied from the software at each recording (0.3–300 Hz).

For the morphological examination, each participant had undergone OCT imaging with the Spectralis HDR + OCT instrument (Heidelberg Engineering GmbH, Heidelberg, Germany). Either horizontal or vertical scans through the fovea or 3D volume scans were obtained. The retinal layers were segmented automatically offline by the software used for the examinations (HEYEX v. 2.5.9; Heidelberg Engineering). The central foveal thickness was considered as the thickness from the pit of the fovea to the outer margin of the retina pigment epithelium (RPE).

### Data analysis and statistics

The averaged ffERG single traces extracted from both eyes for each participant at DA 3 were considered for the offline analyses and imported into MATLAB (R2021b, The MathWorks, Natick, Massachusetts). A time–frequency analysis was performed using a complex Morlet wavelet convolution to extract both time and frequency representation of the ERG signal. The formula and the procedures implemented for the time–frequency analysis are the same described in our previous study [46]. This method allowed to extract different features of the signal, such as frequency (Hz), time (ms) and power (dB, squared of the magnitude). By looking at the changes in power as a function of frequency and time, it is possible to explore in more detail how the OPs evolve. Moreover, the amplitude and time of a- and b-waves calculated by the Espion E2 software were also extracted. For the a-wave, the implicit time (IT) and the amplitude were considered as the deepest trough after the stimulus flash. B-wave’s IT was calculated as the following most positive peak after the a-wave, and its amplitude was measured by adding the a-wave amplitude. Together with age and central foveal thickness, all the parameters were exported as a.csv table and imported subsequently into Python as DataFrame [47] for the statistical analyses.

The power of the OPs and the changes in central foveal thickness was computed between the groups using one-way Analysis of Covariance (ANCOVA) controlling for age. Statistically significant results with a *p*-value smaller than 0.05 were subjected to the Tukey test for post-hoc corrections. IT and amplitude values extracted from a- and b-waves were tested for significance between the groups using Student’s t-test. Given the multiple comparisons, Bonferroni correction was used when the *p*-value was smaller than 0.05. A correlation matrix between the parameters extracted from the ERG’s components (a-wave, b-wave and OPs) was computed with the Pearson’s r and the *p*-values < 0.05 were corrected for multiple comparisons using Bonferroni. All the statistical tests were performed in Python using the Statsmodels package [48].

## Results

### ERG results

**Fig. 1:**
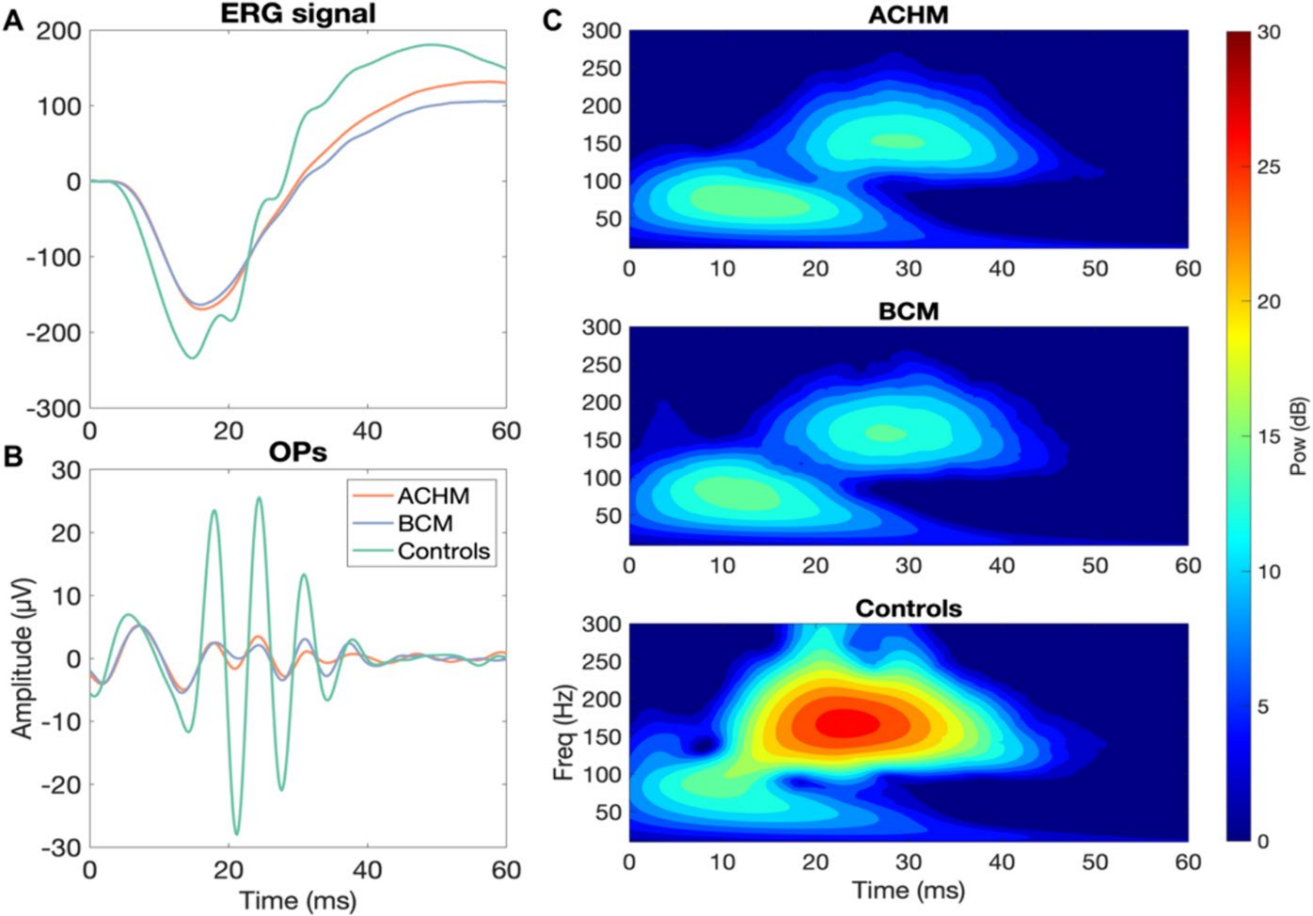
Full-field ERG signal at 3 cd.s.m^−2^. The left panel represents the ffERG traces **a** averaged over all the participants of ACHM, BCM and control groups. Filtered traces (75–300 Hz) of the ffERG signal (**b**) show the oscillatory potentials (OPs) for each group. Right panel **c** shows the time–frequency maps of the averaged ffERG signal of each group depicting the relative power (dB) in the three groups

Figure 1 shows the ERG traces and the filtered signal representing the OPs for each group as well as the time–frequency maps. In the ERG signal, both a- and b-wave amplitudes as well as Ops amplitude are visibly reduced compared to the controls. Such reduction can also be observed in the power shown in the time frequency maps. In the range of frequencies attributed to OPs, it is therefore possible to observe the presence of this component in all groups, although it is largely reduced in the two groups with cone dysfunction.

**Fig. 2:**
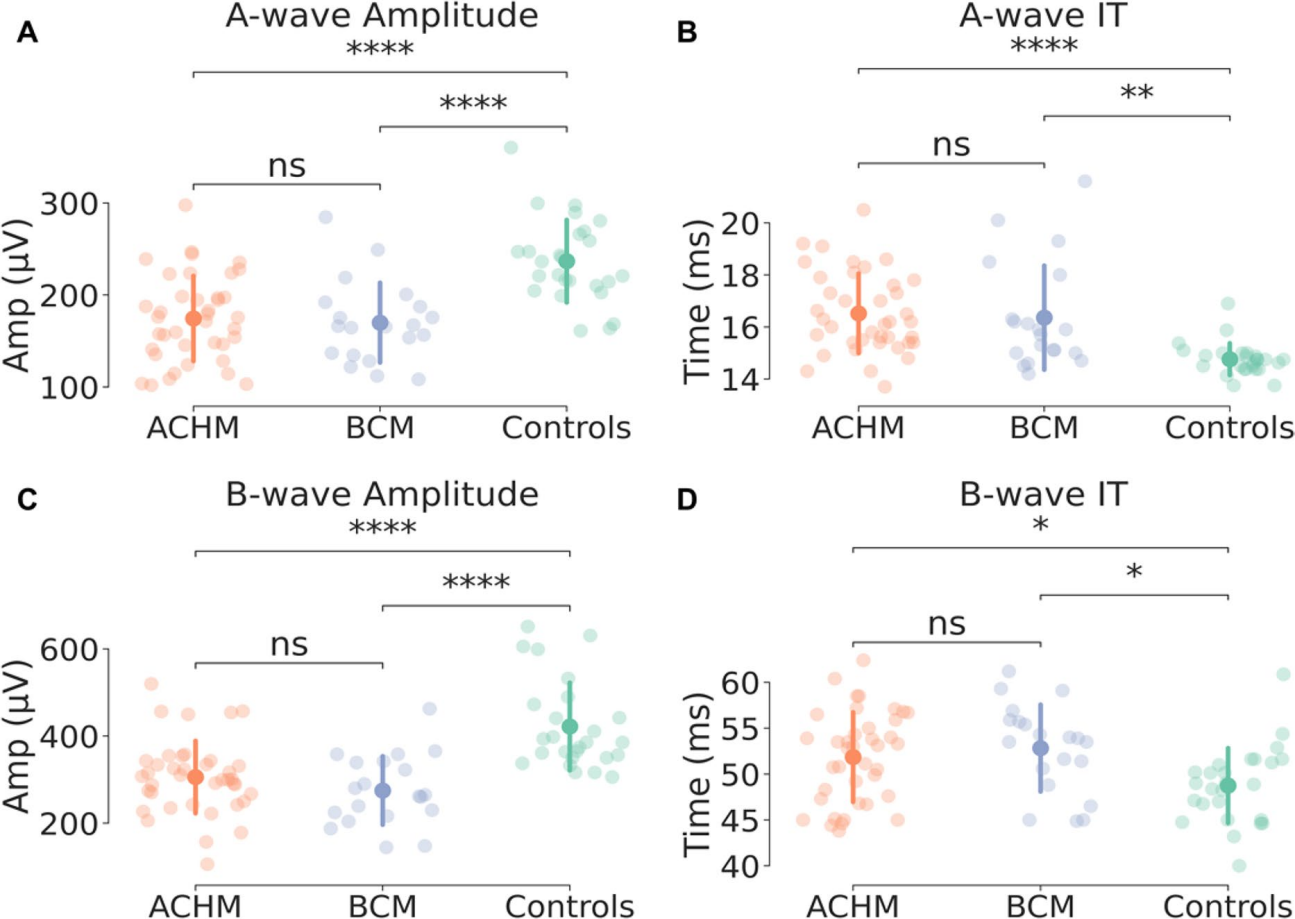
Between groups statistical comparisons between ACHM, BCM and controls of a- and b-wave parameters. Top: a-wave amplitude **a** and IT **b**. Bottom: b-wave amplitude **c** and IT **d**. Asterisks indicate statistically significant differences (*p < .05; **p < .001; **** = *p* < .00001; ns = not significant)

The amplitude and the implicit time of a- and b-waves extracted from the participants were compared between the three groups and are shown in Fig. 2. For the a-wave, the results show a statistically significant difference between ACHM and BCM compared to controls in both parameters, amplitude (ACHM: t-test_(63)_ = 5.287, *p*-corrected < 0.0001, BCM: t-test_(45)_ = 4.962, *p*-corrected < 0.0001) and implicit time (ACHM: t-test_(63)_ = 5.489, *p*-corrected < 0.00001, BCM: t-test_(45)_ = 3.754, *p*-corrected < 0.001). In contrast, no differences were found between the two disease groups. Statistically significant results were also found for the b-wave amplitude between the two groups with cone dysfunction compared to controls (ACHM: t-test_(63)_ = 5.008, *p*-corrected < 0.00001, BCM: t-test_(45)_ = 5.262, *p*-corrected < 0.00001). The IT of b-wave also shows no significant difference between the ACHM and BCM groups, while significant differences were found between the two disease groups in comparison with controls (ACHM: t-test_(63)_ = 2.636, *p*-corrected < 0.05, BCM: t-test_(45)_ = 3.067, *p*-corrected < 0.05).

Regarding the OPs, the parameters corresponding to the peak of the power within time and frequency domains extracted with the time–frequency analysis were compared among the groups. The ACHM and BCM groups did not show statistically significant differences in any of the three parameters considered. However, they did exhibit statistically significant differences compared to the control group only with respect to time and power (time: ACHM: t-test_(63)_ = 3.017, *p*-corrected < 0.05, BCM: t-test_(45)_ = 4.104, *p*-corrected < 0.0001; power: ACHM: t-test_(63)_ = 9.022, *p*-corrected < 0.00001, BCM: t-test_(45)_ = 7.456, *p*-corrected < 0.00001).

**Fig. 3:**
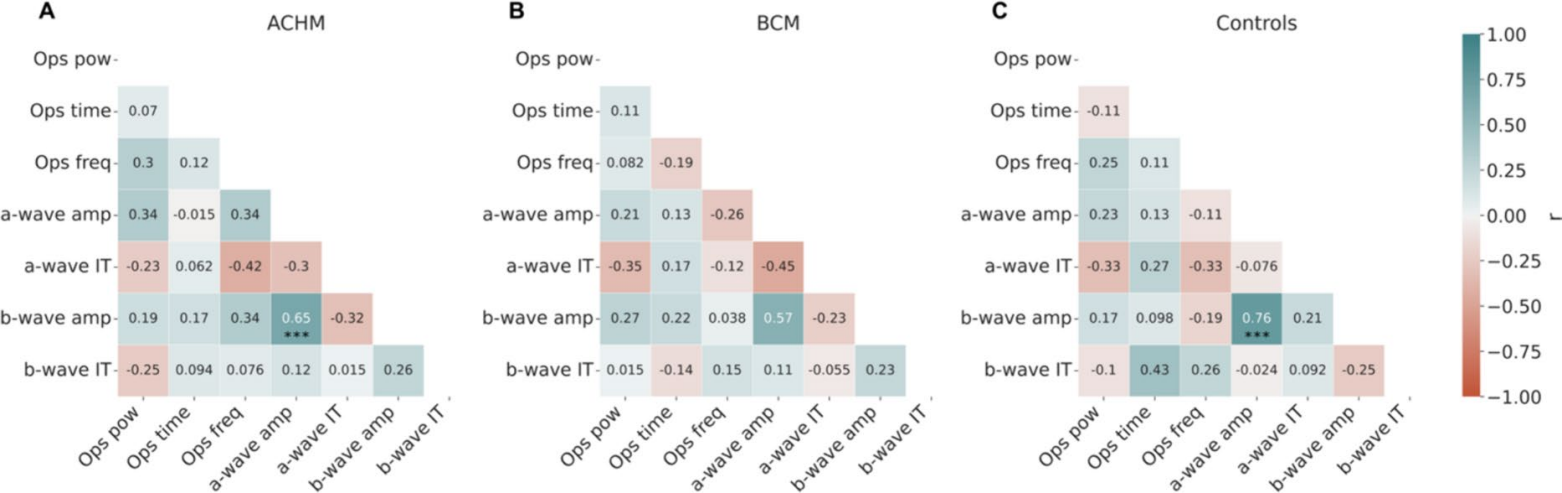
Correlation matrices based on Pearson’s r and its associated p-value corrected for multiple comparisons using Bonferroni method. The correlation between OPs, a- and b-waves parameters are shown for ACHM (**A**), BCM (**B**) and controls (**C**). Significant correlations (*p* < .0001) are indicated with asterisks

The relationships between the extracted electrophysiological parameters are presented in Fig. 3. Specifically, the Pearson’s reported in the correlation matrix provides a quantitative description of the relationships between OPs’ frequency, time and power, and amplitude and IT of the a- and b-wave. Within each group, the significance has been reported and corrected for multiple comparisons using Bonferroni. Overall, a strong correlation was observed between the amplitude of a- and b-waves and was significant in the ACHM (r = 0.65, *p*-corrected < 0.001) and control groups (r = 0.76, *p*-corrected < 0.001). The same tendency, although not significant, was found also for the BCM group. The relationship between OPs and a- and b-waves’ parameters, didn’t show any statistically significant result within the groups. To mention, the b-wave is calculated from the lowest trough of the a-wave to its maximum peak. Consequently, the correlation matrix reports the amplitude values of the b-wave, which also includes the amplitude of the a-wave. However, despite the multicollinearity of these data, the OPs do not appear significantly related to the other ERG parameters.

### Imaging

**Fig. 4:**
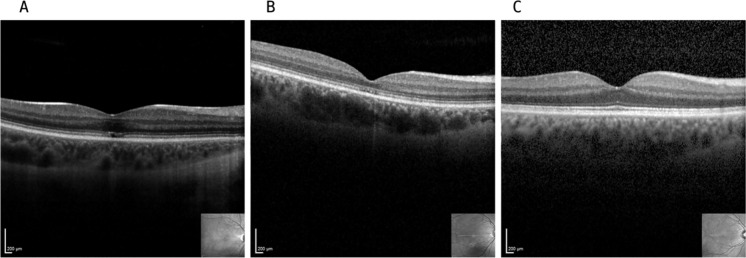
Representative SD-OCT longitudinal sections through the fovea of a ACHM (**A**), BCM (**B**) and a control (**C**) subject

Figure 4 shows SD-OCT scans from representative subjects of each group.

**Fig. 5:**
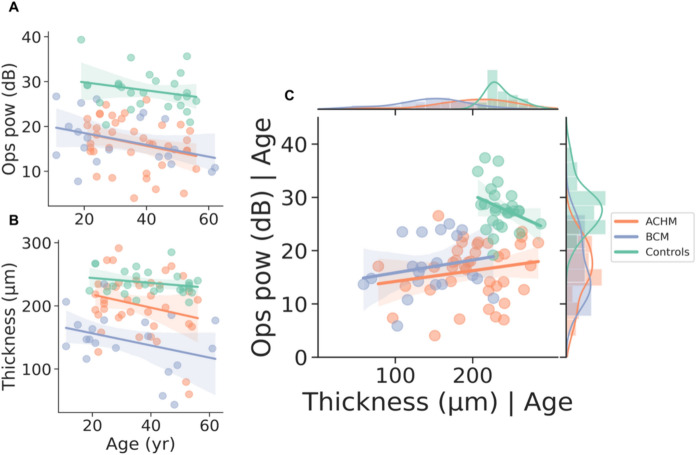
Correlation plots are presented. Panel **a** depicts the correlation between OPs and age, showing non-significant results (*p* > .05) in any of the groups. Panel **b** depicts the correlation between central foveal thickness and age across the three groups, with non-significant results (*p* > .05). In panel c depicts a partial correlation plot indicates the relationships between OPs and thickness when controlling for age in each group, resulted also not significant (*p* > .05). The correlations are based on Pearson’s r value

With the aim of exploring a possible relationship between morphology and retinal function, the power of the OPs extracted from the time–frequency maps and the thickness of the central fovea were correlated in the three groups Fig. 5.

The central foveal thickness is compared between the groups. The results from the one-way ANCOVA showed a significant difference in the central foveal thickness between the three groups after controlling for age (F_(2,81)_ = 29.08, *p* < 0.0001). Tukey post-hoc test confirmed the significance, with the BCM group having a thinner retina compared to ACHM (MD − 56.80, CI [− 84.12, − 29.49], *p* = 0.004) and controls (MD − 91.48, CI [− 121.02, − 61.95], *p* < 0.001), and the ACHM group with a foveal thickness significantly reduced compared to controls (MD − 34.67, CI [− 59.82, − 9.53], *p* < 0.001). No statistically significant correlation between foveal thickness and age was found in any group (Controls: r = − 0.241; ACHM: r = − 0.235; BCM: r = − 0.340).

A statistically significant difference between the three groups was also found by ANCOVA in the OPs' power correcting for age [F_(2,81)_ = 46.785, *p* < 0.0001]. In contrast to foveal thickness, there was no significant difference between the ACHM and BCM group following Tukey’s post-hoc test (MD − 0.64, CI [− 4.11, 2.82], *p* = 0.89). However, both the ACHM and BCM groups differed significantly to the control group (MD − 11.74, CI [− 14.93, − 8.55], *p* < 0.001; MD − 11.10, CI [− 14.85, -7.35], *p* < 0.001, respectively). As for foveal thickness, the relationship between OPs' power and age was not found to be significantly correlated in any of the groups (Controls: r = − 0.242; ACHM: r = − 0.282; BCM: r = − 0.378), although BCM individuals exhibited a trend. Regarding the relationship between morphology and functionality, none of the groups showed a significant correlation as measured by Pearson’s r (Controls: r = − 0.228; ACHM: r = 0.180; BCM: r = 0.196) after correcting for age using a partial correlation. However, the inverse trend of the control group compared to the two groups with cone dysfunction should be noted.

## Discussion

Complete achromatopsia and BCM are two rare genetic diseases with differences in the functionality of the cone subtypes. While in the first, ffERG reveals the total absence of cone function, the latter shows reduced, but recordable photopic responses. In individuals with BCM, L- and M-cone function is erased due to loss of L- and M-photopigment expression, whereas the S-cones remain unaffected. In a prior publication authored by our team [46], we investigated OPs in individuals with ACHM and control subjects. Our findings revealed that despite a significant reduction in OPs, which may be attributed to the absence of cones, they were still discernible. In this study, we examined the influence of the exclusive presence of S-cones in subjects with BCM on the generation of dark-adapted OPs in the full-field Standard 3.0 ERG. Moreover, we investigated potential correlations between the functional and morphological aspects, also considering age dependence.

### Electrophysiological results

The results of this study show that in terms of amplitude and IT parameters of the components elicited by the mixed response of cones and rods (a- and b-waves) at 3 cd.s.m^−2^, ACHM and BCM do not exhibit statistically significant differences. The lack of a significant difference between these two groups suggests that these responses are primarily driven by rods and that S-cones do not contribute or contribute only minimally to the photoreceptor response in the a-wave and to the bipolar cell response in the b-wave for this condition. Additionally, the time and frequency analysis, revealing the evolution of OPs, does not show any significant difference in power between ACHM and BCM. However, in both disease groups OPs are present within the same frequency range as the control group, although they appear significantly delayed in time and reduced in power in comparison to the latter.

In the within-groups correlations of the ERG parameters depicted in Fig. 3, it can be noted that the tendency of such correlations differs in the ACHM and BCM group when compared to the control group. The only statistically significant correlation was observed between a- and b-wave amplitudes in the ACHM and control groups, while BCM followed the same strong trend, albeit not significant. In control conditions, time, frequency, and power parameters of OPs do not appear to correlate with the amplitude and IT of a- and b-waves. OPs are characterized as a phenomenon whose functional mechanisms are still under debate. The lack of a robust correlation among these retinal components suggests that the oscillations observed in the OPs may reflect the involvement of a different retinal network and a potential different stage in information transmission. Regardless of speculative hypotheses about the OPs’ origin, our data suggest that this network may not be primarily linked in a linear fashion with the direct input from photoreceptors, specifically the a-wave.

Previous studies have investigated the hypothesis of involvement of ON and OFF pathways in the generation of the OPs. In the light-adapted condition, the a-wave appears to be mediated not only by the hyperpolarization of photoreceptors but also by the bipolar cells of the OFF pathway [49,50], followed by the depolarization of ON bipolar cells that form the b-wave. Animal studies, using pharmacological interventions, revealed that the manipulation of distinct drugs affecting the ON and OFF pathways inhibits specific components, referred to as early and late, in the context of OPs [45,51]. In the context of dark-adapted mixed rod-cone response, data from our study indirectly support the involvement of ON and OFF pathways in modulating the OPs. In the case of ACHM, the absence of cone function could result in the alteration of these two pathways, causing a significant reduction in the OPs' power. On the other hand, this study demonstrates that the preserved functionality of S-cones in the BCM group does not confer any benefit to the generation of OPs. It is still debated how and to what extent S-cones are linked to the OFF pathway [52,53]. Further investigations are necessary to better assess this assumption.

### OPs and foveal thickness

Our data on the correlation of the power of OPs and the morphological structure of retinal layers within the groups reveal no significant relationship, excluding age as a covariate. These findings showed a statistically significant reduction in central foveal thickness between the BCM and ACHM groups compared to the control group.

Central foveal thickness has been and continues to be used as a useful biomarker for observing the progression and presence of dysfunction. When compared with retinal functionality in ACHM and BCM, however, it may not faithfully mirror a disease. The reduction in thickness in these two groups may, therefore, be confined to the thinning of a specific retinal layer (the outer nuclear layer, i.e., the photoreceptor layer). As reported in previous studies, retinal thinning in ACHM and BCM patients may be linked to structural alterations of cone inner and outer segments 24,26,27, while no information is given for the inner nuclear layer to our knowledge. The relationship between retinal function and morphology proves to be more intricate than the mere correlation of ffERG and central foveal thickness since functional signals reflect a broad area of interconnected cells and networks involved in generating complex responses such as OPs. Unlike other degenerative retinal conditions characterized by the simultaneous functional and morphological degradation of initially functioning photoreceptors, ACHM and BCM experience absence of cone function from birth which persists throughout an individual’s lifetime. It is hence crucial to observe that, despite its stationary course in functions, their degeneration results in a distinctive morphological progression. Consequently, the conventional progression indicator, such as the central retinal thinning, may not be a suitable measure in ACHM or BCM, as the degeneration of photoreceptors does not follow the same course compared to conditions where the cells were initially functional. Understanding this distinction emphasizes the need for tailored methods in the assessment of ACHM and BCM, acknowledging the unique dynamics governing the development of these dysfunctions.

It is important to recognize therefore, that a limiting factor of this retrospective study is the lack of an effective evaluation method targeting the complexity of individual OPs components. Among future directions is the development of an appropriate electrophysiological protocol aimed at investigating the dynamics underlying the OPs component.

## Conclusions

In conclusion, our findings indicate that S-cones do not have a discernible functional influence on the OPs. Furthermore, the observed decrease in the OPs' power cannot be attributed singularly to a reduction in the a-wave, thus suggesting the involvement of a complex non-linear functional network driven by inputs from the photoreceptors. Additionally, the alterations in the morphological structure demonstrated no direct correlation with the observed functional changes. Overall, this study has raised new questions regarding the function and physiological role of the OPs, opening avenues for further exploration and investigation.

## Declarations

### Statement on the welfare of animals

No animals were used in this study.

## References

[1] Bowmaker JK, Dartnall H (1980). Visual pigments of rods and cones in a human retina. J Physiol.

[2] Curcio CA (1991). Distribution and morphology of human cone photoreceptors stained with anti-blue opsin. J Comp Neurol.

[3] Calkins DJ (2001). Seeing with S cones. Prog Retin Eye Res.

[4] Heukamp AS, Warwick RA, Rivlin-Etzion M (2020). Topographic Variations in Retinal Encoding of Visual Space. Ann Rev Vision Sci.

[5] Gouras P, MacKay C (1990). Electroretinographic responses of the short-wavelength-sensitive cones. Invest Ophthalmol Vis Sci.

[6] Stockman A, MacLeod DIA, DePriest DD (1991). The temporal properties of the human short-wave photoreceptors and their associated pathways. Vision Res.

[7] Gouras P (2003). The role of S-cones in human vision. Doc Ophthalmol.

[8] Aboshiha J, Dubis AM, Carroll J, Hardcastle AJ, Michaelides M (2016). The cone dysfunction syndromes. Br J Ophthalmol.

[9] Wissinger B (2001). CNGA3 mutations in hereditary cone photoreceptor disorders. Am J Hum Genet.

[10] Kohl S (2000). Mutations in the CNGB3 gene encoding the β-subunit of the cone photoreceptor cGMP-gated channel are responsible for achromatopsia (ACHM3) linked to chromosome 8q21. Hum Mol Genet.

[11] Johnson S (2004). Achromatopsia caused by novel mutations in both CNGA3 and CNGB3. J Med Genet.

[12] Kohl S (2005). CNGB3 mutations account for 50% of all cases with autosomal recessive achromatopsia. Eur J Hum Genet.

[13] Kohl S (2002). Mutations in the cone photoreceptor G-protein α-subunit gene GNAT2 in patients with achromatopsia. Am J Hum Genet.

[14] Kohl S (2012). A nonsense mutation in PDE6H causes autosomal-recessive incomplete achromatopsia. Am J Hum Genet.

[15] Chang B (2009). A homologous genetic basis of the murine cpfl1 mutant and human achromatopsia linked to mutations in the PDE6C gene. Proc Natl Acad Sci.

[16] Kohl S (2015). Mutations in the unfolded protein response regulator ATF6 cause the cone dysfunction disorder achromatopsia. Nat Genet.

[17] Gardner JC (2014). Three different cone opsin gene array mutational mechanisms; genotype-phenotype correlation and functional investigation of cone opsin variants. Human Mutation.

[18] Wissinger B (2022). The landscape of submicroscopic structural variants at the OPN1LW/OPN1MW gene cluster on Xq28 underlying blue cone monochromacy. Proc Natl Acad Sci.

[19] Gardner JC (2009). Blue cone monochromacy: causative mutations and associated phenotypes. Mol vision.

[20] Hirji N, Aboshiha J, Georgiou M, Bainbridge J, Michaelides M (2018). Achromatopsia: clinical features, molecular genetics, animal models and therapeutic options. Ophthalmic Genet.

[21] De Silva SR (2021). The X-linked retinopathies: Physiological insights, pathogenic mechanisms, phenotypic features and novel therapies. Prog Retin Eye Res.

[22] Young R, Price J (1985). Wavelength discrimination deteriorates with illumination in blue cone monochromats. Invest Ophthalmol Vis Sci.

[23] Michaelides M (2005). Blue cone monochromatism: a phenotype and genotype assessment with evidence of progressive loss of cone function in older individuals. Eye.

[24] Thomas MG, Kumar A, Kohl S, Proudlock FA, Gottlob I (2011). High-resolution in vivo imaging in achromatopsia. Ophthalmology.

[25] Patterson EJ (2022). Foveal cone structure in patients with blue cone monochromacy. Invest Ophthalmol Vis Sci.

[26] Barthelmes D (2006). Quantitative analysis of OCT characteristics in patients with achromatopsia and blue-cone monochromatism. Invest Ophthalmol Vis Sci.

[27] Cideciyan AV (2013). Human cone visual pigment deletions spare sufficient photoreceptors to warrant gene therapy. Hum Gene Ther.

[28] Genead MA (2011). Photoreceptor structure and function in patients with congenital achromatopsia. Investig Opthalmol Visual Sci.

[29] Zobor D (2017). The clinical phenotype of CNGA3-related achromatopsia: pretreatment characterization in preparation of a gene replacement therapy trial. Investig Opthalmol Visual Sci.

[30] Sundaram V (2014). Retinal structure and function in achromatopsia. Ophthalmology.

[31] Andreasson S, Tornqvist K (1991). Electroretinograms in patients with achromatopsia. Acta Ophthalmol.

[32] Moskowitz A, Hansen RM, Akula JD, Eklund SE, Fulton AB (2009). Rod and rod-driven function in achromatopsia and blue cone monochromatism. Invest Opthalmol Visual Sci.

[33] Perlman, I. (2007) The Electroretinogram: ERG. In: Webvision., Kolb H, Nelson R, Fernandez E, Jones B, (eds) The Electroretinogram: ERG, The organization of the retina and visual system

[34] Penn R, Hagins W (1969). Signal transmission along retinal rods and the origin of the electroretinographic a-wave. Nature.

[35] Stockton RA, Slaughter MM (1989). B-wave of the electroretinogram. A reflection of ON bipolar cell activity. J General Physiol.

[36] Miller R, Dowling J (1970). Intracellular responses of the Müller (glial) cells of mudpuppy retina: their relation to b-wave of the electroretinogram. J Neurophysiol.

[37] Yonemura D, Masuda Y, Hatta M (1963). The oscillatory potential in the electroretinogram. Jpn J Physiol.

[38] Wachtmeister L (1987). Basic research and clinical aspects of the oscillatory potentials of the electroretinogram. Doc Ophthalmol.

[39] Robson AG (2022). ISCEV Standard for full-field clinical electroretinography (2022 update). Doc Ophthalmol.

[40] Kizawa J, Machida S, Kobayashi T, Gotoh Y, Kurosaka D (2006). Changes of oscillatory potentials and photopic negative response in patients with early diabetic retinopathy. Jpn J Ophthalmol.

[41] Eggers ED, Carreon TA (2020). The effects of early diabetes on inner retinal neurons. Vis Neurosci.

[42] HennyHeynen LW, van Norren D (1985). Origin of the oscillatory potentials in the primate retina. Vision Res.

[43] Wachtmeister L (1998). Oscillatory potentials in the retina: what do they reveal. Progr Retinal Eye Res.

[44] Liao F, Liu H, Milla-Navarro S, Villa PDL, Germain F (2023). Origin of retinal oscillatory potentials in the mouse, a tool to specifically locate retinal damage. Int J Mol Sci.

[45] Dong C-J, Agey P, Hare WA (2004). Origins of the electroretinogram oscillatory potentials in the rabbit retina. Vis Neurosci.

[46] Righetti G (2021). Oscillatory potentials in Achromatopsia as a tool for understanding cone retinal functions. Int J Mol Sci.

[47] McKinney W (2010) Data structures for statistical computing in python. In: Proceedings of the 9th python in science conference, vol 445, Austin, TX, pp 51–56

[48] Seabold S, Perktold J (2010) Statsmodels: econometric and statistical modeling with python. In: Proceedings of the 9th python in science conference, vol 57l. Austin, TX, pp 10–25080

[49] Bush RA, Sieving PA (1994). A proximal retinal component in the primate photopic ERG a-wave. Invest Ophthalmol Vis Sci.

[50] Robson JG, Saszik SM, Ahmed J, Frishman LJ (2003). Rod and cone contributions to the a-wave of the electroretinogram of the macaque. J Physiol.

[51] Wachtmeister L (1980). Further studies of the chemical sensitivity of the oscillatory potentials of the electroretinogram (ERG) I. GABA- and glycine antagonists. Acta Ophthalmol (Copenh).

[52] Klug K, Herr S, Ngo IT, Sterling P, Schein S (2003). Macaque retina contains an S-cone OFF midget pathway. J Neurosci.

[53] Lee SCS, Telkes I, Grünert U (2005). S-cones do not contribute to the OFF-midget pathway in the retina of the marmoset, *Callithrix jacchus*. Eur J Neurosci.

